# The effectiveness of dietary intervention in osteoarthritis management: a systematic review and meta-analysis of randomized clinical trials

**DOI:** 10.1038/s41430-025-01622-0

**Published:** 2025-04-28

**Authors:** Sara Asadi, Sara Grafenauer, Claire V. Burley, Caroline Fitzgerald, Peter Humburg, Belinda J. Parmenter

**Affiliations:** 1 UNSW Medicine & Health Lifestyle Clinic, School of Health Sciences, Faculty of Medicine & Health, UNSW Sydney, Sydney, NSW 2052 Australia; 2 Department of Exercise Physiology, School of Health Sciences, Faculty of Medicine & Health, UNSW Sydney, Sydney, NSW 2052 Australia; 3 Department of Nutrition, Dietetics and Food Innovation, School of Health Sciences, Faculty of Medicine & Health, UNSW Sydney, Sydney, NSW 2052 Australia; 4 Dementia Centre of Excellence, enAble Institute, Curtin University, Perth, WA Australia; 5 Mark Wainwright Analytical Centre, UNSW Sydney, Sydney, NSW 2052 Australia; 6 School of Health, University of the Sunshine Coast, Sippy Downs, QLD Australia

**Keywords:** Nutrition, Rheumatic diseases

## Abstract

This study aimed to evaluate the impact of various dietary interventions on managing osteoarthritis (OA), a condition significantly affecting global health due to joint alterations driven by inflammatory mediators. A systematic review and meta-analysis, adhering to PRISMA guidelines, examined Randomized Controlled Trials (RCTs) investigating dietary interventions in OA. Two reviewers independently conducted study selection, data extraction, and quality assessment. Random effects models calculated standardized mean differences (SMD) and mean differences (MD). Risk of bias was evaluated with the Cochrane Risk of Bias tool (RoB2), and heterogeneity was assessed using I² values. Nine RCTs (898 participants) were identified, assessing various diets: reduced energy (*n* = 4), Mediterranean (*n* = 2), low-fat (*n* = 2), anti-inflammatory (*n* = 1), low-carbohydrate (*n* = 1), and plant-based (*n* = 1). Dietary interventions significantly improved pain (SMD: –0.67; 95% CI: [–1.01, –0.34]; *p* < 0.0001), and physical function (SMD: –0.62; 95% CI: [–0.94, –0.30]; *p* = 0.0001) and body weight (MD: –3.18; 95% CI: [–3.52, –2.83], *p* < 0.0001). Subgroup analyses revealed reduced energy diets improved pain (SMD: –0.85; 95% CI: [–1.15, –0.55], *p* < 0.0001), physical function (SMD: –0.95; 95% CI: [–1.33, –0.58], *p* < 0.0001) and body weight (MD: –3.13; 95% CI: [–3.77, –2.49], *p* < 0.0001). The Mediterranean diet did not significantly impact pain (SMD: –0.27; 95% CI: [–1.14, 0.60], *P* = 0.54), or physical function (SMD = –0.28; 95% CI: [–0.79, 0.24], *p* = 0.29). This study emphasizes the significant impact of dietary interventions on pain, physical function, and weight management in people with OA, with reduced energy diets showing the most effectiveness. Specific dietary patterns show promise but require further investigation.

## Introduction

Osteoarthritis (OA) is a prevalent and persistent joint condition that significantly impacts the overall wellbeing and quality of life of individuals worldwide [1]. As of 2020, around 595 million people were diagnosed with OA globally, and if the present patterns continue, it is estimated that close to one billion individuals will experience OA by the year 2050 [2]. In the initiation and progression of the OA process, inflammatory mediators play a vital role. These mediators are produced locally by joint cells and, systemically, by tissues like adipose tissue, which releases them into the bloodstream [3]. Eventually, they reach the joint through the vasculature in the subchondral bone. Consequently, these mediators induce diverse structural and functional alterations in joint tissues, including the breakdown of cartilage, remodelling of bone, and changes in the synovium [3]. These alterations lead to noticeable symptoms such as pain, stiffness, swelling, and limitations in joint mobility. OA, being one of the most widespread chronic health concerns, goes beyond physical ramifications. It influences mental well-being, disrupts sleep patterns, hinders work engagement, and, notably, can even impact mortality [4].

With the incidence of OA on the rise due to the aging population and the obesity epidemic, there is a growing need to prioritize effective prevention and management of this chronic condition. Recent research highlights the potential role of dietary factors in preventing the onset or slowing the progression of OA [5, 6]. One noteworthy avenue of investigation involves the adoption weight management strategies, which have shown promising outcomes. Studies reveal that a reduction of at least 5–10% of body weight correlates with significant improvements in OA-related symptoms and functional outcomes [7, 8]. This suggests a tangible link between weight management and the amelioration of OA, presenting a compelling case for dietary interventions [8].

An expanding body of research is delving into the potential efficacy of anti-inflammatory diets, such as the Mediterranean Diet (Med Diet) and plant-based diets, as interventions for OA. These dietary patterns are characterized by an emphasis on fruits, vegetables, whole grains, and healthy fats, have garnered attention for their potential to influence the inflammatory processes underlying OA [9, 10]. Empirical evidence indicates notable efficacy of anti-inflammatory diets in reducing inflammation, irrespective of concurrent weight loss, as substantiated in academic literature [11, 12], suggesting this might be promising for OA.

Drawing insights from a comprehensive systematic review and meta-analyses, it becomes evident that a synergistic approach involving reduced energy diets and regular exercise yields a moderate alleviation of pain [7]. Another meta-analysis has highlighted the positive impact of incorporating meal replacements into a dietary regimen, revealing improvements in physical function [13]. Additionally, a separate systematic review reveals that dietary modifications can effectively reduce pain and enhance function in adults with osteoarthritis [14]. However, it is noteworthy that the existing body of evidence from systematic reviews and meta-analyses primarily focuses on the combination of a reduced energy diet and exercise, and specifically on interventions involving meal replacements. There is a notable gap in the literature regarding the efficacy of different dietary patterns such as the Med diet, anti-inflammatory diets, and distinct reduced energy dietary strategies without meal replacement on OA management. As such, there is a compelling need for further investigation to systematically assess and compare the outcomes associated with these diverse dietary approaches in the context of OA management. This study represents a significant advancement in the field by being the first meta-analysis to systematically compare the effectiveness of diverse dietary patterns in OA management, extending beyond the focus on energy restriction and exercise. By evaluating the impact of anti-inflammatory, low-carbohydrate, low-fat, and Mediterranean diets, this work fills a critical gap in the literature by focusing solely on the effects of diet without the combined influence of exercise or meal replacement formulas. Also, it can provide evidence-based insights that have the potential to shape future research directions. The primary aim of this systematic review and meta-analysis was to examine the effects of various dietary interventions, sourced from Randomized Controlled Trials (RCTs), on pain relief, weight management, and physical function in individuals with OA, offering a more comprehensive understanding of dietary strategies in OA management.

## Methods

The systematic review and meta-analysis protocol were pre-registered on the Open Science Framework (Registration 10.17605/OSF.IO/CYHWP). There were minor deviations from the pre-registered protocol. Specifically, the quality assessment tool. These were addressed by using the Cochrane Risk of Bias tool (RoB 2), the updated tool for quality assessment in RCTs. This revised tool RoB2 was developed to address limitations identified in the other tools, incorporate advances in the assessment of risk of bias used in other recently developed tools, and integrate recent developments in the estimation of intervention effects from randomized trials [15, 16]. Adherence to reporting standards was ensured by following the Preferred Reporting Items for Systematic Reviews and Meta-Analyses (PRISMA) guidelines [17].

### Search strategy

In conducting this systematic review and meta-analysis, we identify relevant studies by systematically searched the following electronic databases from their inception until January 2024: PubMed (Medline), EMBASE, Web of Science, CINAHL, PsycINFO, Scopus, Central Register of Controlled Trials, and grey literature. Additionally, we set up automated updates in PubMed to track newly published papers, with the most recent update received on January 29, 2025. To ensure comprehensive coverage, we also conducted manual searches in other databases to capture any newly available studies. The search strategy was developed with input from an information librarian, ensuring the comprehensiveness and accuracy of the search process. This ensured that the strategy was comprehensive and aligned with the study’s objectives. The detailed search strategy for all databases is provided in Supplementary Appendix 1. We did not impose any language restrictions on the search and provided a translator when required. Further, we screened reference lists of the included studies, and systematic reviews on the topic.

### Study selection

We included RCTs studies that recruited male and female adults (over 18 years of age) who were diagnosed with OA. OA is a chronic joint condition characterized by the breakdown and gradual loss of cartilage in the joints. It typically has the potential to affect any joint within the body, although it typically has a stronger presence in joints located in the hands, knees, hips, and spine [18]. This systematic review did not impose restrictions on the joints affected.

Studies that report any diet strategies including any type of diet intervention like weight loss diet, low fat diet, Med diet, low carbohydrate diet, anti-inflammatory diet, and plant-based diet for the management of pain, physical function, quality of life or other health outcomes in patients with OA were included. Diet strategies were classified based on the diet reported in each study. Health outcomes included body weight, and inflammatory markers such as C-reactive protein (CRP), interleukin-1(IL-1), interleukin-6 (IL-6), and tumour necrosis factor-alpha (TNF-α).

Studies carried out in healthcare settings, communities, and homes were included. There were no limitations based on geographic location or cultural context. Inclusion criteria for RCTs involved direct comparisons between different diets or a usual care control group. If an exercise component was provided in both the intervention and control groups, and the effect of the dietary intervention could be isolated, then these studies were included.

Studies combining diet and meal replacement formulas, longitudinal study designs, analytical observational studies, such as prospective and retrospective cohort studies, case-control studies, analytical cross-sectional studies, and qualitative literature, surveys, case series, studies without full text and case reports were excluded.

### Screening strategy

The authors followed the strict screening procedures reported by the Cochrane Handbook for Systematic Reviews. Articles were exported from databases into COVIDENCE software (Australia, ABN 41 600 366 274). Screening of titles and abstracts to remove irrelevant reports was undertaken by one author (SA), as permitted by the Cochrane Handbook, which states that while duplicate screening is ideal, initial screening by a single reviewer is acceptable [19]. Articles or documents that met the inclusion criteria without meeting any exclusion criteria were retrieved for full text review. The full texts of these selected articles and documents were then evaluated by two authors (SA & BP). Each author operated independently and remained blind to each other’s decision throughout the screening process. Regarding reviewer blinding, while researchers have historically recommended blinding reviewers to reduce potential bias, there is currently no empirical evidence to demonstrate that blinding significantly reduces or prevents bias [20]. Consequently, reviewers in this study were not blinded to author identities, institutional affiliations, or other details, which aligns with common practice in systematic reviews. Instead, we minimized bias through clearly defined inclusion and exclusion criteria, adherence to systematic procedures, and independent assessment by multiple reviewers [20]. Discrepancies during the full-text screening phase were addressed through discussion, consistent with Cochrane’s recommendation [19] that disagreements are often resolved through dialogue to identify simple oversights or differences in interpretation. When necessary, a third reviewer (SG) provided arbitration to reach a final decision. Multiple publications arising from the same study were linked together, and the publication most relevant to the research question was selected as the primary source, with secondary papers used for data resources if needed.

### Data extraction

Two authors (SA & CF) worked independently to extract data from the included studies. Disagreements were resolved by a discussion. If consensus was not reached, a third author (SG) was consulted. The following information was decided upon a-priori and extracted from included papers: study characteristics including first author, year of publication, country, study design, sample size, study duration, adherence, participants’ characteristics such as body mass index (BMI), sex, race, study population characteristics (e.g., overweight, pre-diabetic state, or type 2 diabetes), intervention and control conditions such as type of diets, details of the diet intervention and control group, changes in outcomes such as changes in weight, BMI, pain, physical function, quality of life (QoL), mental health, and inflammatory markers including CRP, IL-1, IL-6, and TNF-α.

If the mean and standard deviation (SD) of the differences were unavailable, our initial step was to attempt to communicate with the study authors. In the event of no response, we proceeded by extracting the mean and SD of both pre-intervention and post-intervention values for the control and intervention groups. Authors were contacted twice for missing data. If no response was received within one month of initiating contact, and both the mean and SD values for pre-intervention and post-intervention were unavailable, the paper was considered to meet the inclusion criteria but lacked sufficient data for the meta-analysis.

### Quality assessment

The studies included were assessed for bias using the Rob2 tailored for RCTs [16]. Two independent reviewers evaluated each study to ascertain whether it exhibited low, moderate, or high risk of bias. Evaluation criteria encompassed potential biases stemming from subject recruitment, randomization procedures, intervention deviations, missing data handling, outcome measurement, and reported result selection. The two reviewers resolved any disagreements about bias and quality assessments by discussing them. If consensus was not reached, a third author was consulted.

### Data synthesis and statistical analysis

The statistical analysis was conducted using R software^TM^ (Indianapolis, Indiana, USA) where the mean difference, SD, and sample size were entered. When a study did not report SD, we used the estimation methods recommended by the Cochrane Handbook [21]. If the difference values were not directly available, the differences were computed using below equation:

1 $${\rm{SD}}.\,{{\rm{diff}}}^{2}={({\rm{SD\; baseline}})}^{2}+{({\rm{SD\; end}})}^{2}-2{\rm{R}}\times {\rm{SD\; baseline}}\times {\rm{SD\; end}}$$

assuming a correlation coefficient (R value) of 0.8 [22].

If the standard error (SE) was provided in the article, SD was calculated using below equation:

2 $${\rm{SD}}={\rm{SE}}\surd {\rm{n}}$$

with n representing the sample size [21].

We analysed continuous data by calculating the mean difference (MD) (meta-analysis of the same scale) or standardized mean difference (SMD) (meta-analysis of different scales), and 95% confidence intervals.

Forest plots were utilized to visually represent the point estimate and the 95% CI of each individual effect size. Effect sizes were interpreted according to Cohen’s interpretation ‘trivial’ (<0.20), ‘small’ ( ≥ 0.20 to <0.50), ‘moderate’ ( ≥0.50 to <0.80) and ‘large’ ( ≥ 0.80) [23]. The heterogeneity of data between studies was evaluated and quantified using Cochrane’s Q test, with a *p*-value of <0.1 indicating statistical significance, and I^2^ [24]. The Cochrane Handbook provides general guidelines for interpreting heterogeneity levels and a random-effects meta-analysis was used to incorporate heterogeneity among studies. Heterogeneity between 0% and 40% might not be deemed important, while between 30% and 60% may suggest moderate heterogeneity. Heterogeneity between 50% and 90% may indicate substantial heterogeneity, and between 75% and 100% may represent considerable heterogeneity [25]. In cases where a study’s I^2^ value fell into two intervals, we opted for the higher, more conservative interval. We conducted meta-regression analyses to investigate how continuous factors, such as the length of follow-up periods, could impact the results. The p-value of regression coefficient was used to indicate whether this difference was statistically significant [26]. Sensitivity analyses were performed using leave-one-out meta-analyses to evaluate the impact of each individual study on the overall estimate of the treatment effect. Additionally, we conducted a sensitivity analysis by removing the single study with ‘low risk’ of bias to assess whether its inclusion was disproportionately affecting the results. The meta-regression and sensitivity were conducted using R software.

## Results

### Search Results

**Fig. 1:**
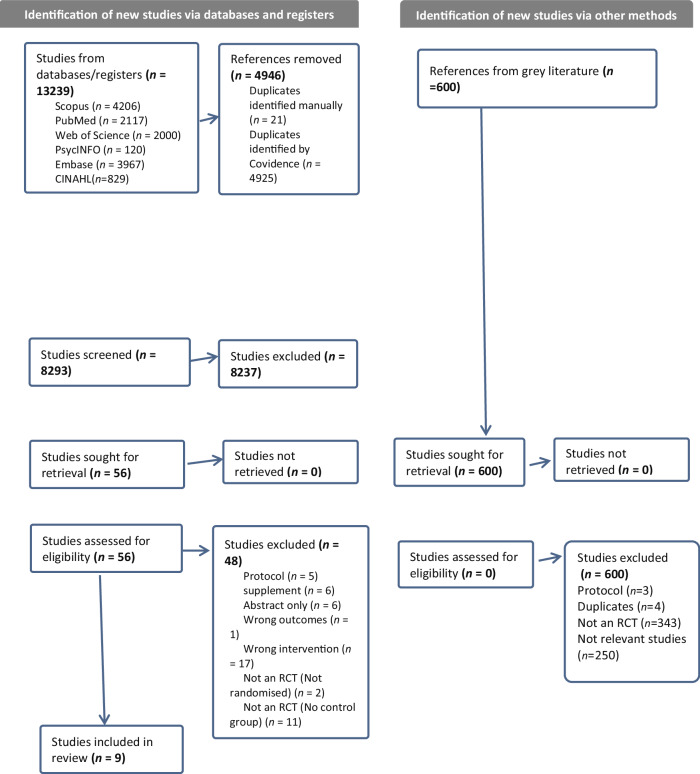
Preferred reporting items for systematic reviews and meta-analysis (PRISMA) flow diagram for study selection.

Figure 1 shows the PRISMA flow diagram of the study selection process. The search identified 13,239 records. Of these, 8293 were screened after removal of duplicates, and 8237 were excluded based on titles and abstracts. Of 56 potentially relevant records screened in full text for eligibility (Supplementary Appendix 2), nine RCTs (*n* = 898) met our inclusion criteria [7, 8, 27–33]. Additionally, Supplementary Appendix 3 provides a detailed summary of how each PRISMA 2020 checklist item is addressed within the manuscript, ensuring transparency and adherence to reporting guidelines.

### Study characteristics

Participant and intervention characteristics are outlined in Tables 1, 2. All nine RCTs encompass a diverse geographic distribution, with contributions from the United States (*n* = 4), Iran (*n* = 2), Tunisia (*n* = 1), Taiwan (*n* = 1), and the United Kingdom (*n* = 1). The studies encompassed a total of 898 participants, with sample sizes across the studies varying from 21 to 316 individuals. The age of participants included in the studies ranged from 41 ± 11.7 to 72 ± 1.97 years old. The study population comprised individuals with a BMI range from 26.9 ± 3.02 to 36 ± 7 kg/m^2^. Additionally, four studies included patients with OA who also had other comorbidities such as diabetes mellitus, cardiovascular diseases, hyperlipidaemia, liver disease, and kidney failure. Regarding gender diversity, seven of the studies included participants of all genders, one study specifically focused on female participants, and one study did not specify gender.

**Table 1:** Characteristics of included studies.

Authors, year, country	Study Design	Language published	Population Inclusion Criteria	Sample Size	Race	Sex (% female)	Other Comorbidities	Age (year)	BMI Mean ± SD	Trial Duration (weeks)	Outcomes	Findings
Messier et al. [7], USA	RCT	English	Overweight and obese (BMI ≥ 28) with knee OA	316	23% non white	71%	48% hypertension 26% CVD 8% diabetes	69 ± 7.1^a^ 69 ± 6.9^b^	34.2 ± 5.3^a^ 34 ± 6.1^b^	72	WOMAC pain Physical function SF-36 Weight	While both dietary weight-loss-only and exercise-only interventions demonstrated effectiveness in enhancing specific outcomes, it is evident that the combined intervention consistently yielded the most positive impact on the pain, physical function, weight and health related quality of life.
Clinton et al. [27], USA	Prospective Randomized Unblinded Trial	English	Knee OA	40	8.2% non-white	83.8%	29.7% hypertension 35.1% depression	60 ± 6.3^a^ 56 ± 8.4^b^	28.4 ± 4.5^a^ 29.1 ± 6.5^b^	6	SF-36 Weight BMI VAS pain	A whole-foods, plant-based diet significantly improves pain, weight, physical function, and BMI.
Dyer et al. [29], UK	RCT	English	OA	99	NA	82.8%	Patients with other comorbidities were excluded.	60 ± 12^a^ 66 ± 11^b^	NA	16	Pain Physical Function Serum biomarkers (IL-1α, IL-2, IL-6, IFN-γ, TNFα)	There were no differences between groups in the response of any AIMS2 components and most biomarkers, except the pro-inflammatory cytokine IL-1α in individuals with OA.
Strath et al. [32], USA	RCT Pilot	English	Knee OA	21	28.5% African- American 66.6% non-Hispanic white 4.7% Hispanic	57%	Patients with other comorbidities were excluded.	68 ± 7.1^a^ 72 ± 1.9^b^ 71 ± 3.1^b^	26.9 ± 3.02^a^ 26.6 ± 4.48^b^ 35.6 ± 7.3^b^	12	Weight BMI BPI pain KOOS pain KOOS QoL CRP IL-6 IFN-γ TNFα	There was an improvement in QOL, self-reported pain in adults with OA following the Low Carbohydrate Diet
Hsu et al. [8], Taiwan	RCT	English	Obese (BMI of 27-35) with knee OA	66	NA	63.4%	63.4% used statins.	64 ± 4.1^a^ 65 ± 3.9^b^	30.8 ± 2.47^a^ 31.1 ± 2.6^b^	12	WOMAC Pain Weight BMI Physical function	The combination of an individual diet control intervention along with telemedicine-based resistance exercise intervention led to a notable enhancement in body composition, blood biochemistry, and lower-limb functional performance among the studied population with concurrent health conditions. Top of Form
Sadeghi et al. [31], Iran	Randomized Feeding trial	English	Overweight and obese (25 < BMI < 35) with knee OA	129	NA	91.2%	None	59 ± 9.8^a^ 57 ± 10^b^ 55 ± 9.5^b^	NA	12	Weight Pain Physical function	The Med diet group experienced a decrease in pain severity, while patients following low-fat and regular diets did not show a significant change in pain levels. The MD and low-fat diet groups exhibited a significantly greater reduction in weight.
Dolatkhah et al. [28], Iran	RCT parallel	English	Overweight and obese (BMI of 25-40) with knee OA	60	NA	100%	6.6% CVD 1.6% diabetes mellitus 28.3% hypertension 1.6% hyperlipidaemia 3.3% liver disease 5% kidney failure	54 ± 8.1^a^ 52 ± 6.7^b^	34.5 ± 5.58^a^ 34.9 ± 5.48^b^	8	Pain Physical function QOL Weight Anxiety Depression	The combination of an anti-inflammatory agent with a low-calorie diet led to more substantial weight loss and greater enhancements in pain intensity, functional status, depression, anxiety, and certain aspects of quality of life among overweight and obese women with knee osteoarthritis, as compared to the low-calorie diet alone.
Wolf et al. [33], USA	RCT Single blinded	English	Overweight and obese (27 ≤ BMI ≤ 40) with knee OA	111	11% non-white	13%	NA	69 ± 8.9^a^ 67 ± 7.4^b^	33 ± 5^a^ 36 ± 7^b^	24	Physical Function Weight	Individuals in the two groups that received nutritional counselling experienced a higher level of weight loss and compared to participants in groups without nutritional counselling.
Ghroubi et al. [30], Tunisia	RCT Prospective	French	Severe obesity (BMI ≥ 35) or moderate obesity (30 ≤ BMI < 35) with knee OA	56	NA	NA	Patients with at least one risk factor (diabetes, dyslipidemia, hypertension, hyperuricemia) were included.	42 ± 9.8^a^ 41 ± 11.7^b^	39.2 ± 3.7^a^ 38.7 ± 6.15^b^	104	Pain Weight	Integrating weight loss with exercise leads to greater enhancements in physical function and pain relief for obese adults suffering from knee OA than either strategy applied separately.

*PGIC* Patient Global Impression of Change, *CES-D* Centre for Epidemiologic Studies Depression Scale, *VAS* Visual Analogue Scale, *AIMS2* Arthritis Impact Measurement; Scale, *WOMAC* Total Western Ontario and McMaster Universities Arthritis Index, *BDI-II* Beck Depression Inventory, *BAI-II* Beck Anxiety Inventory, *SF-36* Short Form 36 Health Survey Questionnaire, *CES-D* Centre for Epidemiologic Studies Depression Scale, *RCT* Randomized Controlled Trial, *BMI* Body Mass Index, *CVD* Cardiovascular Diseases, *NA* not available.

^a^Control group.

^b^Intervention group.

**Table 2:** Characteristics of intervention and control groups.

Authors, year, country	Intervention Diet (Details)			Groups	Control (Details)	Dietary adherences, %
	Diet (Type)	More details	Recommendations			
Messier et al. [7], USA	Reduced Energy Diet	Achieve a 5% average weight loss	Aim was to raise awareness regarding the significance of modifying eating habits to reduce caloric intake.	1-Dietary weight loss plus Exercise 2-Exercise 3-Dietary weight loss 3-Healthy lifestyle (control)	Social interaction and health education on topics concerning osteoarthritis, obesity, and exercise.	72
Clinton et al. [27], USA	Whole Food Plant-Based	No restriction in energy intake. Derive a minimum of 90% of their calories from plant sources.	Using fruits, vegetables, unrefined foods, legumes, and grains, with a strict avoidance of animal products.	1-Whole Food Plant-Based 2-Control	Usual care	NA
Dyer et al. [29], UK	Mediterranean Diet	Dietary advice was provided consistent with a Mediterranean diet.	NA	1-Mediterranean 2-Control	Usual care	65
Strath et al. [32], USA	1-Low Carbohydrate Diet	20 g daily total carbohydrate intake, with the option to increase it to 40 g. There were no restrictions on fats or protein (from meats and eggs).	Fruits were restricted, and vegetables were allowed in restricted amounts.	1-Low Carbohydrate Diet 2-Low Fat Diet (weight loss) 3-Control	Eat as usual Educational documents related to portion control	NA
	2-Low Fat Diet	Males had a daily calorie reduction of 500 kcal, while females had a reduction of 250–300 kcal. 60% of daily calories from carbohydrates, 20% from protein, and 20% from fats.	High in fruits, vegetables, low-fat foods, whole grains, low-fat dairy, and limited cholesterol and saturated fats			
Hsu et al. [8], Taiwan	Reduced Energy Diet	A balanced low-energy diet of 1200 kcal/day	An individualized nutritional plan for each participant was designed	1- Resistance Exercise Group 2- Resistance Exercise Group plus Diet Control Group 3- Diet Control Group	Resistance Exercise Group	83
Sadeghi et al. [31], Iran	1-Mediterranean. (28 kcal/kg/day)	35% from fats, 50% from carbohydrates, and 15% from protein. 27–37 grams/day of fibre, prioritize whole grains, limit red meat to 150 grams/ month.	Patients receive mercury-free fish oil supplements twice a week. Use olive oil for salads and canola oil for frying. The diet includes a daily serving of legumes and nuts, permits low-fat dairy, and recommends drinking six glasses of water each day	1-Mediterranean 2-Low-fat diet 3-Control	Usual care	lower than 80% were excluded
	2-Low fat diet. (28 kcal/kg/day)	20% of total daily calories from fats, 65% from carbohydrates, and 15% from protein.	In this dietary pattern, patients did not receive any advice and dietary serving were determined for each patient.			
Dolatkhah et al. [28], Iran	1-Anti-inflammatory plus Reduced Energy Diet	Fresh fruits and vegetables, constituting two-thirds of the total caloric intake per meal. Protein is derived from plant sources, and fish and low-fat dairy. Carbohydrates are sourced from fruits, vegetables, and whole grains.	Plant-based fats, such as alpha-linolenic acid from flaxseed and walnuts, are encouraged, while trans-fatty acids should be avoided. Olive oil is the preferred cooking oil. Using turmeric, garlic, ginger, and cinnamon. Omega-3 fatty acids can be added to the diet as a supplement.	1-Anti-inflammatory 2-Reduced Energy Diet	Reduced Energy Diet	64.8% women reported being adherent every day. 25.9%women reported being adherent most days. 9.3% women reported being adherent sometimes
	2-Reduce Energy Diet	500 kcal below individual energy requirements. less than 30% of calories from fat, 55–60% from carbohydrates, and 10–15% from protein.	After introducing the diet, each participant received explanations about the food pyramid, the substitution tables, and the role of each food group.			
Wolf et al. [33], USA	Reduced Energy Diet	The modification of eating habits was personalised, taking into account individual lifestyles.	Providing information about dietary fat intake and proper proportions of vegetables	1-Home-Based Exercise 2-Weight Control Nutritional Programme 3-Home-Based Exercise plus Weight Control Nutritional Programme 4-Usual care	Usual care	NA
Ghroubi et al. [30], Tunisia	Reduced Energy Diet	Reduction of caloric intake by 25-30% for participants whose habitual food consumption is between 1500-3000 calories	No additional information was provided.	1-Exercise only 2- Diet plus exercise 3- Diet only 4- Control group	Usual care	NA

The diets encompassed in the study varied, including reduced energy (*n* = 4) [7, 8, 30], Med Diet (*n* = 2) [29, 31], low-fat (defined as 20% fat) (*n* = 2) [31, 32], anti-inflammatory (*n* = 1) [28], low-carbohydrate (defined as 20 g/d for first three weeks then 40 g thereafter) (*n* = 1) [32], and whole-foods, plant-based diet (*n* = 1) [27]. Messier et al. described reduced energy diet as achieving a 5% reduction in body weight through calorie reduction [7]. Another study defined it as adhering to a balanced low-energy diet of 1200 kcal/day (1 kcal = 4.186 kJ) [8]. Ghroubi et al. characterized it as reducing caloric intake by 25–30% for individuals whose usual consumption ranges from 1500 to 3000 calories [30]. Wolf et al. referred to it as weight control through modified eating habits [33]. Therefore, we propose renaming all these approaches collectively as a “reduced energy diet.” The length of time the dietary interventions ran varied significantly across the trials, with a mean duration of 29.55 ± 34.46 weeks, range 6 to 104. Control/comparator groups across the studies received different interventions, including eat-as-usual (*n* = 6), a low-calorie diet (*n* = 1), and health education sessions covering topics related to OA, obesity, and exercise (*n* = 1). Compliance with the intervention was evaluated using various dietary assessment methods, such as weekly food journals, 24-h recalls, and 7-day food diaries, in seven of the papers [7, 8, 27–29, 31, 32]. However, two studies did not specify the method used to assess compliance [30, 33]. The RCTs investigated several outcome measures, including pain (*n* = 9), weight reduction (*n* = 8), physical function (*n* = 7), QoL (*n* = 3), and inflammatory markers (*n* = 2). However, we could not perform a meta-analysis on QoL and inflammatory outcomes due to the insufficient number of studies and data. All the studies, with the exception of Wolf et al. [33], reported findings on pain. All studies reported on body weight change, with the exception of Dyer et al. [29]. Two studies did not report on physical function [27, 32].

### Risk of bias (Rob)

**Fig. 2:**
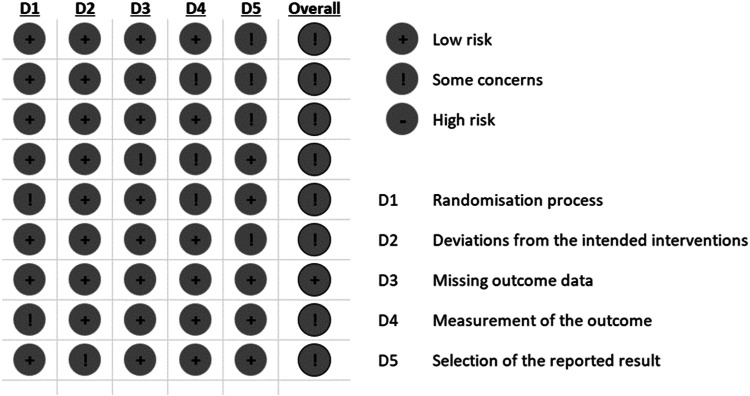
Risk of bias assessment using the revised Cochrane risk-of-bias (RoB 2).

Figure 2 presents an overview of the risk of bias within the studies, which were evaluated based on the pre-established criteria of the Cochrane Rob2 tool for randomized control [16].

Within Domain 1: Randomization Process, seven studies were identified as having a low risk of bias [7, 8, 27–31], while the other three studies were classified as having some concerns of bias [32, 33]. In Domain 2: Deviations from Intended Interventions, every study was assessed to have a low risk of bias except Ghroubi et al. [30]. One study had some concerns of bias for Domain 3: Missing outcome data [27], and the remainder had a low risk of bias. In Domain 4: Measurement of the outcome, six studies had low risk of bias [7, 8, 28–30, 33] and three had some concerns of bias [27, 31, 32]. Five studies [7, 27, 30, 32, 33] had low risk of bias for Domain 5: Selection of the reported result, with the remainder having some concerns of bias [8, 28, 29, 31]. Overall, analysis showed some concerns with risk of bias for the eight studies included in this meta-analysis.

### Changes in pain

**Fig. 3:**
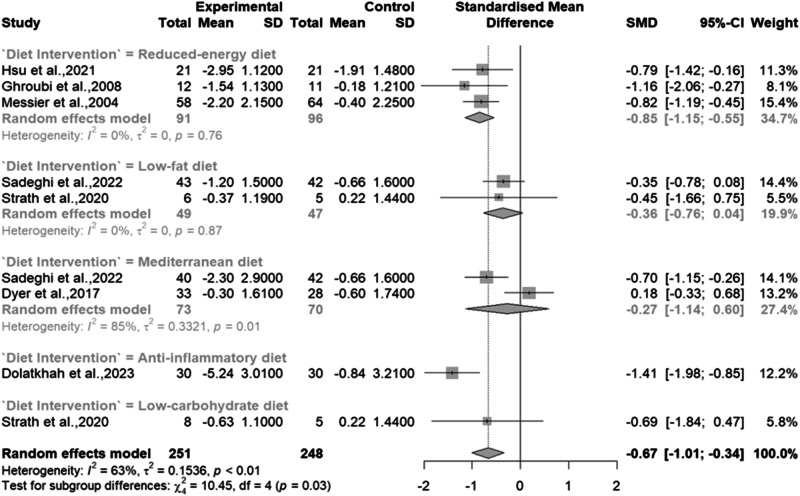
Meta-analysis of the effects of dietary interventions on Pain.

Across the seven studies [7, 8, 28–32] that measured subjective pain via questionnaire, there was a significant improvement in subjective pain levels, with the experimental groups demonstrating moderate and significant improvements compared to the control groups (SMD -0.67; 95% CI: [–1.01, –0.34]; *p* < 0.0001). However, there was notable heterogeneity (I^2^ = 63%, *p* < 0.01) among the studies [Fig. 3].

When analysing by sub-groups, a reduced energy diet led to a large, significant improvement in pain (SMD: –0.85; 95% CI: [–1.15, –0.55], *p* < 0.0001), with zero heterogeneity (I^2^ = 0%; *p* = 0.76). However, no significant improvement was observed for the low-fat diet (SMD: –0.36; 95% CI: [–0.76, 0.04], *p* = 0.08) and Med Diet (SMD: –0.27; 95% CI: [–1.14, 0.60], *p* = 0.54) diets. Heterogeneity for the low-fat diet (I^2^ = 0%, *p* = 0.87) was low, but high and significant for the Med Diet (I^2^ = 85%, *p* = 0.01). Due to a limited number of studies, we were unable to complete a sub-group analysis for the anti-inflammatory or low carbohydrate diets, however the anti-inflammatory diet showed promising results with a large and significant reduction in pain (SMD –1.41; 95% CI: [–1.98, –0.85], *P* < 0.0001).

### Changes in physical function

**Fig. 4:**
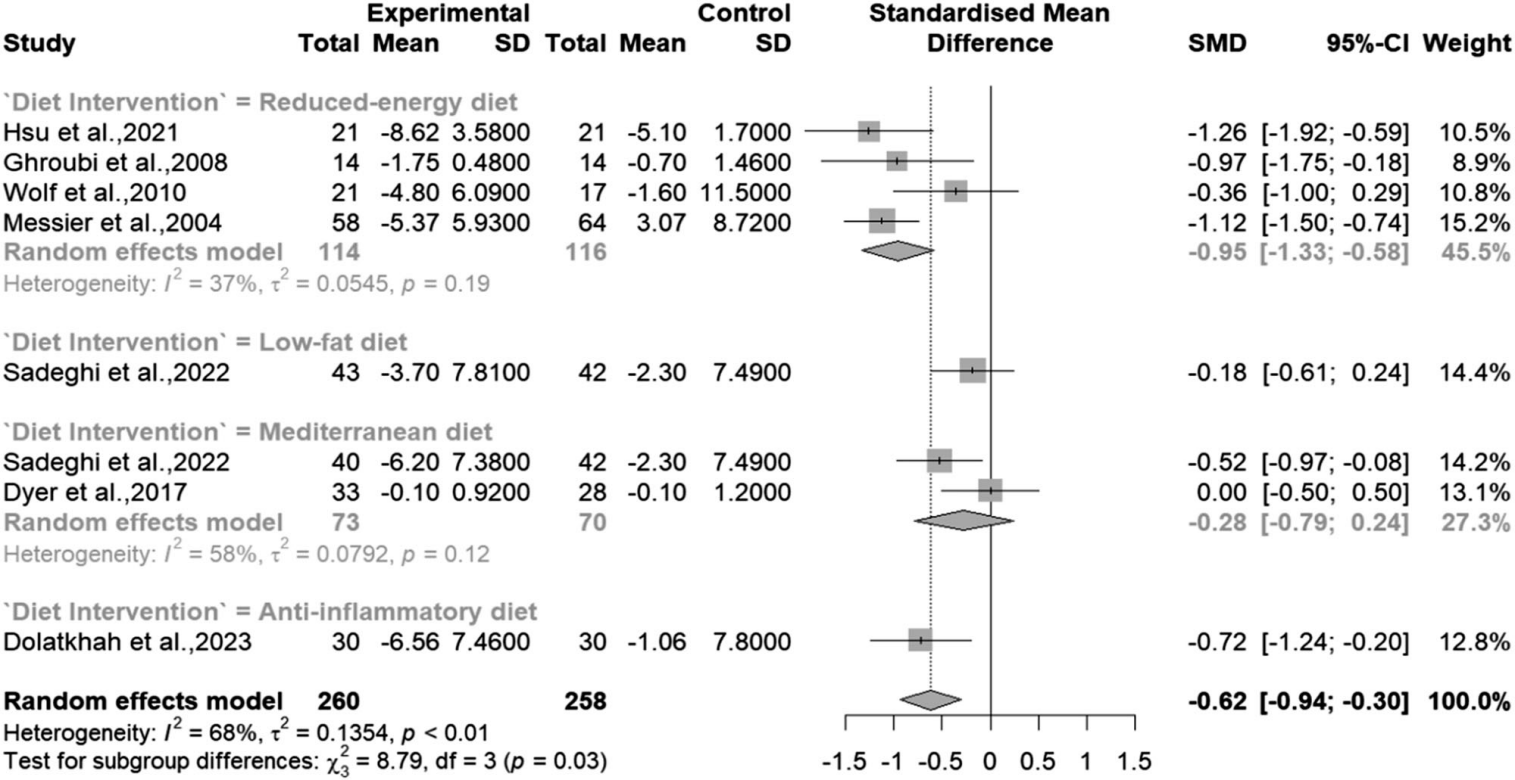
Meta-analysis of the effects of dietary interventions on Physical function.

In the seven studies [7, 8, 28–31, 33] that assessed subjective physical function using questionnaires, there was a significant moderate improvement in physical function overall, with groups undergoing experimental treatments showing better outcomes than control groups (SMD: –0.62; 95% CI: [–0.94, –0.30]; *p* = 0.0001). However, significant heterogeneity (I^2^ = 68%, *p* < 0.01) was observed across the studies [Fig. 4].

Sub-group analysis revealed that a reduced energy diet led to a large and significant improvement in physical function (SMD: –0.95; 95% CI: [–1.33, –0.58], *p* < 0.0001) with moderate heterogeneity (I^2^ = 37%; *p* = 0.19). In contrast, the Med diet had a small, non-significant effect (SMD: –0.28; 95% CI: [–0.79, 0.24], *p* = 0.29) with notable, but non-significant heterogeneity (I^2^ = 58%, *p* = 0.12). We were unable to conduct a subgroup analysis for the anti-inflammatory diet due to limited study numbers. However, this diet type displayed promising results, indicating a significant and noteworthy improvement in physical function (SMD: –0.72; 95% CI: [–1.24, –0.20], *p* < 0.0001).

### Changes in body weight

**Fig. 5:**
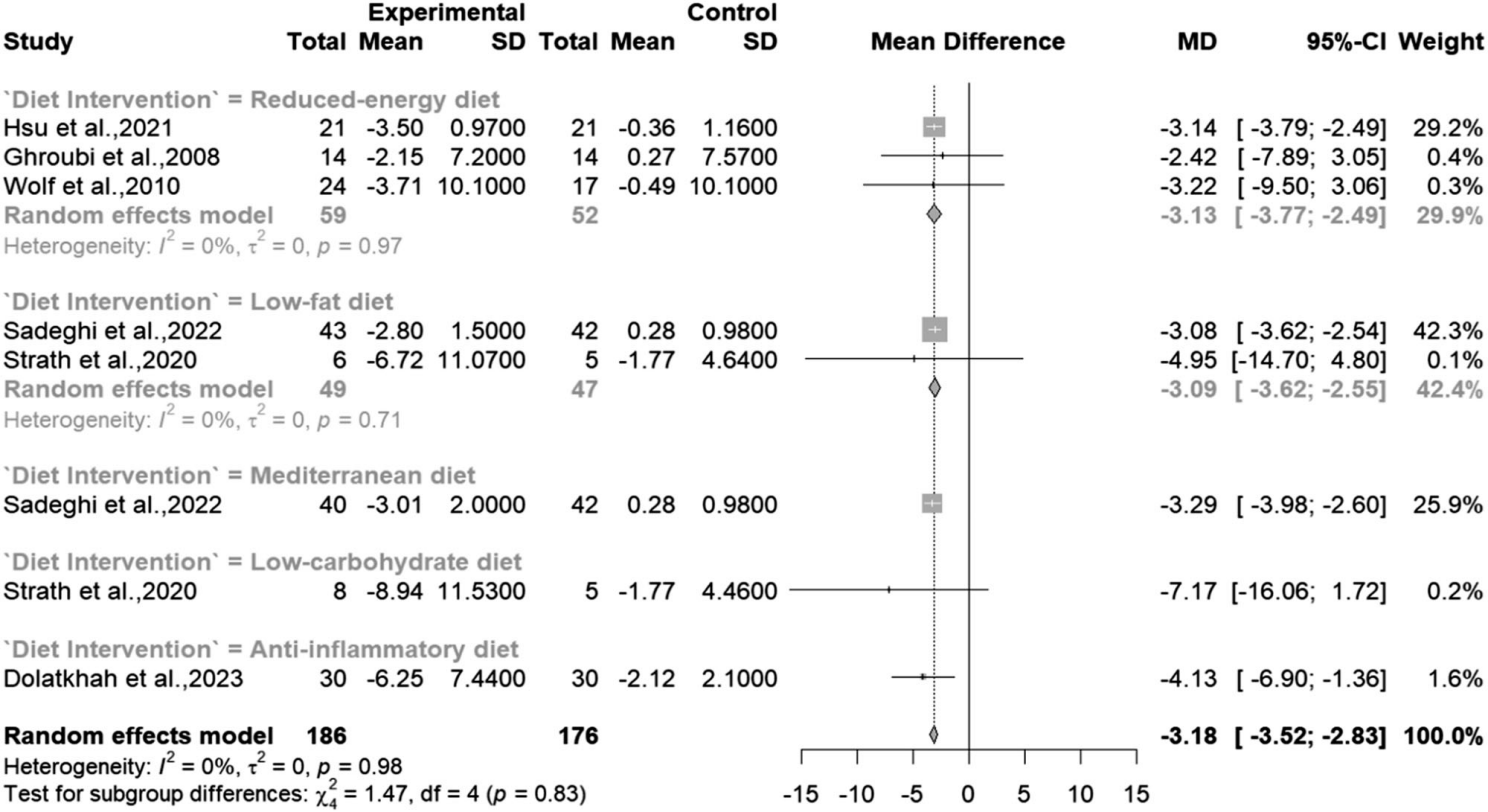
Meta-analysis of the effects of dietary interventions on body weight.

Overall, there was a statistically significant body weight loss across six studies [8, 28, 30–33], with those in the intervention groups showing better outcomes (MD: –3.18; 95% CI: [–3.52, –2.83], *p* < 0.0001) with a low heterogeneity (I^2^ = 0%, *p* = 0.98) across the studies [Fig. 5].

Sub-group analysis showed statistical differences in outcomes for those on a reduced energy diet (MD: –3.13; 95% CI: [–3.77, –2.49], *p* < 0.0001) and low-fat diet (MD: –3.09; 95% CI: [–3.62, –2.55], *p* < 0.0001). There was no heterogeneity among studies for the reduced energy diet (I^2^ = 0%, *p* = 0.97) and low-fat diet interventions (I^2^ = 0%, *p* = 0.71). Due to the scarcity of research, we could not carry out a subgroup analysis for studies using the anti-inflammatory diet, Med Diet, or low-carbohydrate diet. Nonetheless, both the Med Diet (SMD: –3.29; 95% CI: [–3.98, –2.60], *p* < 0.0001), and anti-inflammatory diet (SMD: –4.13; 95% CI: [–6.90, –1.36], *p* < 0.0001), showed promising results.

### Sensitivity analyses

To assess the influence of individual studies on the overall pooled efficacy estimate for the primary outcome, we conducted leave-one-out meta-analyses. In this approach, each study was sequentially removed, and the meta-analysis was recalculated to determine its impact on the pooled effect size.

The leave-one-out analyses demonstrated a consistent pooled effect size, indicating the robustness of the findings. The sensitivity analyses for pain, weight reduction, and physical function demonstrated that no single study significantly influenced the overall results. These findings underscore the robustness of the intervention’s impact across all three outcomes. Detailed results of the leave-one-out analyses can be found in Supplementary Appendix 4.

We also conducted a sensitivity analysis by excluding the study with ‘low risk’ of bias to assess its impact on the results. After removal, the pooled effect sizes remained consistent for both primary outcomes. For physical function, the SMD was –0.52 (95% CI: [–0.83, –0.21]; I² = 54%), and for pain, the SMD was –0.65 (95% CI: [–1.04, –0.26]; I² = 66%). These results further confirm the robustness of the intervention’s effect.

### Meta-regression

To explore how continuous variables, such as the duration of follow-up periods, might influence the outcomes, we conducted meta-regression analyses. We found no significant impact on pain (β = −0.0043, SE = 0.0056, *p* = 0.442), body weight (β = −0.0098, SE = 0.0301, *p* = 0.74), or physical function (β = −0.0071, SE = 0.0047, *p* = 0.126). We were unable to calculate meta regression on other variables due to a limited number of studies.

## Discussion

This systematic review and meta-analysis revealed that dietary interventions have a noteworthy impact on improving pain levels, enhancing physical function, reducing body weight in patients with OA. Specifically, we found that reduced energy diets resulted in significant improvements in pain, physical function, and body weight. However, no significant enhancements in pain and physical function were observed with the Med Diet. To our knowledge, this is the first meta-analysis examining the efficacy of various dietary interventions in managing OA.

Our research indicates that dietary interventions are effective in alleviating pain among patients with OA, with reduced energy diets showing particularly promising results. Consistent with our findings, a meta-analysis revealed that a combination of diet and exercise treatments moderately reduced pain. However, diet-only treatments did not yield pain reduction [34]. It is important to highlight that while our study focused on dietary interventions without the inclusion of meal replacement formulas, the meta-analysis encompassed trials involving a combination of reduced energy diet and formula. However, when subgroup analyses were conducted, the same level of efficacy was not observed with low-fat diets or the Med Diet. A systematic review of three studies (comprising two cohort studies and one trial) concluded a possible association between OA and adherence to the Med Diet [9]. However, our meta-analysis included only two studies on the Med diet, one of which lacked sufficient details about the intervention. Additionally, a significant limitation across the included studies was the method used to assess dietary adherence, which may have affected the reliability of the findings [29]. The high heterogeneity observed in some subgroups, such as the Mediterranean diet, suggests significant variability in study outcomes, which may reflect differences in study design, population characteristics, or intervention protocols. Additionally, the wide confidence intervals for some standardized mean differences (e.g., [-1.42; -0.16] for the reduced-energy diet) indicate uncertainty in the effect estimates. These findings, coupled with the significant subgroup differences (*p* = 0.03), highlight the need for caution in interpreting the results and underscore the importance of further research to clarify the effectiveness of specific dietary interventions in OA. Anti-inflammatory and low carbohydrate diets showed promising results for improving pain, however with only one study available to analyse for each diet type, the literature would benefit from further research utilising these diets.

Furthermore, our research highlights the potential of dietary interventions to enhance physical function among individuals with OA, with particularly positive outcomes associated with reduced energy diets. Aligned with our findings, a systematic review and meta-analysis revealed that incorporating dietary changes alongside meal replacements, and very low-energy diets using meal replacements exclusively, led to improvements in physical function [13]. In contrast, the Med Diet did not demonstrate a notable improvement in physical function. However, due to the inclusion of only two studies on the Med Diet in this meta-analysis, it was challenging to thoroughly examine this relationship. Despite the apparent effectiveness of anti-inflammatory and low-fat diets in alleviating physical function-related issues, the scarcity of studies focusing on these dietary interventions underscores the necessity for additional research in this area.

In relation to weight, the collective findings from seven studies indicated a statistically significant body weight reduction. Subgroup analysis emphasized notable differences in outcomes, especially among individuals following reduced energy or low-fat diets. Across the studies analyzed, treatment durations varied widely, spanning from 6 to 104 weeks. Our investigation involved conducting a meta-regression analysis to probe the influence of treatment duration and our findings revealed no notable impact on pain levels, body weight, or physical function. The evidence suggests that weight reduction may offer a plausible explanation for the decrease in pain. Specifically, in overweight individuals, a reduction of 5% in body weight can alleviate certain joint discomfort, whereas a loss of at least 10% in body weight is associated with substantial clinical improvement in joint pain [34, 35]. Obesity is considered a chronic inflammatory condition, with IL-1 contributing to the susceptibility to knee OA. Consequently, obesity and increased body fat play a significant role in the development of OA, even in non-mechanical contexts. Therefore, weight loss not only reduces mechanical stress on the joints but also mitigates the inflammatory processes associated with obesity, further contributing to the reduction of OA symptoms [36]. In the initiation and progression of OA, inflammatory mediators are pivotal [37, 38]. Managing weight and reducing the consumption of proinflammatory food components can potentially decrease the incidence of chronic inflammation [39]. Various food components may possess anti-inflammatory effects; for instance, polyunsaturated fatty acids (PUFAs) can hinder the production of nitric oxide and TNF-α, while vitamins C and E may mitigate systemic inflammation by reducing oxidative stress and the expression levels of proinflammatory cytokines [40]. Enhancing the intake of food components with a lower Dietary Inflammatory Index (DII), such as those advocated in the Med Diet, can help maintain the body in a more anti-inflammatory state [39].

The current study possesses both strengths and limitations. Notably, it is the first meta-analysis of randomised controlled trials investigating the impact of various dietary strategies on OA management. A thorough search of six databases employing MeSH terms, broad scope, and comprehensive keyword strategy was conducted to encompass the entirety of existing literature and the study refrained from imposing restrictions based on language or time frame. Also, the adoption of RoB 2 ensured a more robust and systematic framework for evaluating potential biases, aligning with contemporary standards in systematic reviews and meta-analyses. The modification provided a clearer and more precise categorization of bias levels while maintaining consistency with the overall trends and conclusions derived from the included studies. This consistency in findings supports the validity of our results and mitigates concerns about potential bias introduced by this change [15, 16]. Additionally, there were certain constraints to consider. A known limitation of diet trials is the issues in blinding participants undergoing interventions. Another limitation arises from the significant heterogeneity observed across the included studies. To address this, we utilized random-effects models to account for variability between studies. Subgroup analyses based on dietary intervention type indicated consistent results with low heterogeneity (I² = 0%) for reduced energy and low-fat diets. Additionally, meta-regression was conducted to evaluate the impact of study duration, but it did not identify duration as a significant source of heterogeneity. The limited number of studies per subgroup restricted further exploration of other potential sources of heterogeneity. According to the Cochrane Handbook, meaningful regression analyses typically require at least ten studies for each characteristic being modelled. This becomes even more critical when covariates are unevenly distributed across studies. Given the small number of included studies in our review, findings from subgroup analyses or meta-regression should be interpreted with caution. Future research with larger datasets is necessary to provide more definitive insights into the factors contributing to heterogeneity.

Furthermore, it is important to note that several included studies did not provide comprehensive data on dietary adherence, complicating the interpretation of the results. Specifically, while seven studies monitored compliance, two studies lacked adherence data entirely. This gap introduces uncertainty about the extent to which participants adhered to the prescribed interventions, which may have affected the reliability of the findings. We also emphasize the need for future research to prioritize standardized and transparent reporting of compliance to ensure more robust and reliable conclusions. Another significant limitation is the variability in the types of reduced energy diet interventions used across different studies. These variations can lead to differing outcomes in terms of weight loss and its impact on OA management. For instance, the effects of a 5% weight reduction might not be directly comparable to those achieved through a fixed 1200 kcal/day diet or a 25–30% caloric reduction. Each of these dietary strategies may influence OA outcomes differently due to variations in the degree of calorie restriction, nutritional balance, and participant compliance. To improve the quality and comparability of future research, it is essential to standardize the definitions and reporting of dietary interventions. Also, we now note that the differences in baseline characteristics, such as age, BMI, and comorbidities, could potentially influence how different populations respond to dietary interventions, thus affecting the generalizability of our results. While this variability adds breadth to the analysis, it also suggests that the findings may not be equally applicable to all subgroups. Future research should aim to investigate the impact of these factors more thoroughly to help tailor dietary recommendations to specific populations. While this study focused solely on the effects of dietary interventions without the inclusion of exercise or meal replacement formulas, we recognize the potential for synergistic effects when diet and exercise are combined. Future research should explore the cumulative impact of these combined interventions on OA outcomes, as there is evidence that integrated approaches involving both dietary modifications and exercise may provide more substantial benefits in alleviating pain and improving physical function [7, 13].

In conclusion, our systematic review and meta-analysis points to a significant impact of dietary interventions on pain relief, enhanced physical function among OA patients. Specifically, reduced energy diets emerged as particularly effective in improving pain levels, physical function, and body weight. Although the Med Diet did not exhibit significant benefits in pain relief or physical function improvement, highlighting the need for further research in this area. Additionally, the scarcity of studies on anti-inflammatory and low-carbohydrate diets calls for more investigation to fully understand their potential in OA management.

## Supplementary information

Appendix 1

Appendix 2.

Appendix 3.

Appendix 4

## Data Availability

The data from this study are available upon request to the corresponding author.
