# Supplementary material for: The effectiveness of dietary intervention in osteoarthritis management: a systematic review and meta-analysis of randomized clinical trials
Source: Eur J Clin Nutr. 2025 Apr 28;79(10):959–71. doi: 10.1038/s41430-025-01622-0 (PMC12537491; doi:10.1038/s41430-025-01622-0)
Supplement: Supplementary file 1 — Appendix 1 [file 41430_2025_1622_MOESM1_ESM.docx]

**Appendix 1:** Detailed Search Strategies

|  | **PubMed Search** |
| --- | --- |
|  | "Osteoarthritis"[Mesh] |
|  | osteoarthritis [Title/Abstract] OR arthritis [Title/Abstract] OR joint degenerative disease [Title/Abstract] OR Cartilage [Title/Abstract] |
|  | diet*[Title/Abstract] |
|  | "Diet"[Majr] |
|  | quality of life[Title/Abstract] OR wellbeing[Title/Abstract] OR weight*[Title/Abstract] OR Symptoms[Title/Abstract] OR Inflame*[Title/Abstract] OR CRP[Title/Abstract] OR C-reactive protein[Title/Abstract] OR interleukin-1[Title/Abstract] OR interleukin-6[Title/Abstract] OR Tumour Necrosis Factor alpha[Title/Abstract] OR IL-1α[Title/Abstract] OR Cytokines[Title/Abstract] OR pain[Title/Abstract] |
|  | **Embase Search** |
|  | exp osteoarthritis/th [Therapy] |
|  | (osteoarthritis or arthritis or joint degenerative disease or Cartilage).ti,ab. |
|  | exp diet/ |
|  | Diet*.ti,ab. |
|  | cytokine/ or inflammation/ or "quality of life"/ |
|  | (quality of life or wellbeing or weight* or symptoms or Inflame* or C-reactive protein or interleukin-1 or interleukin-6 or Tumour Necrosis Factor alpha or Cytokines or pain).ti,ab. |
|  | **Scopus Search** |
|  | osteoarthritis OR arthritis OR "joint degenerative disease" OR cartilage |
|  | Diet |
|  | "quality of life" OR wellbeing OR weight* OR symptoms OR inflame* OR "c-reactive protein" OR interleukin-1 OR interleukin-6 OR "tumour necrosis factor alpha" OR il-1α OR cytokines OR pain |
|  | **Web of Science Search** |
|  | (TI=(osteoarthritis OR arthritis OR joint degenerative disease OR cartilage)) OR AB=(osteoarthritis OR arthritis OR "joint degenerative disease" OR cartilage) |
|  | (TI=(Diet*)) OR AB=(Diet*) |
|  | (TI=(quality of life OR wellbeing OR weight* OR Symptoms OR Inflame* OR CRP OR C-reactive protein OR interleukin-1 OR interleukin-6 OR Tumour Necrosis Factor alpha OR IL-1α OR Cytokines OR pain)) OR AB=(quality of life OR wellbeing OR weight* OR Symptoms OR Inflame* OR CRP OR C-reactive protein OR interleukin-1 OR interleukin-6 OR Tumour Necrosis Factor alpha OR IL-1α OR Cytokines OR pain) |
|  | **PsycINFO Search** |
|  | (Degenerative polyarthritis or "Osteoarthritis of hip" or Osteoarthritis Knee or Generalized osteoarthritis or Osteoarthritis Spine or Thoracic osteoarthritis or Hand osteoarthritis or "Osteoarthritis of ankle" or Idiopathic osteoarthritis or Thumb osteoarthritis or "Osteoarthritis of wrist" or Primary osteoarthritis or "Osteoarthritis of elbow" or "Osteoarthritis of glenohumeral joint" or Nodal osteoarthritis or Osteoarthritis aggravated or Interspinous osteoarthritis or Trapeziometacarpal osteoarthritis or "Osteoarthritis of foot joint" or "Osteoarthritis of toe joint" or "Osteoarthritis of finger joint" or Lumbar Osteoarthritis or Secondary osteoarthritis or Temporomandibular joint osteoarthritis or Osteoarthritis deformans endemica).mp. |
|  | (osteoarthritis or arthritis or joint degenerative disease or Cartilage).mp. |
|  | (Diet or Reducing diet or Elimination Diets or Vegan diet or "Foods and Diets category" or Ketogenic Diet or Vegetarian diet or Diet therapy or Diet education or Normal diet or Diet Healthy or Medical diet or Therapeutic diet or Diet Elemental or Diet Mediterranean or Protein diet or Research diet or Carbohydrate diet).mp. |
|  | Diet*.mp. |
|  | (quality of life or wellbeing or weight* or symptoms or Inflame* or C-reactive protein or interleukin-1 or interleukin-6 or Tumour Necrosis Factor alpha or Cytokines or pain).mp. [mp=title, abstract, heading word, table of contents, key concepts, original title, tests & measures, mesh word] |
|  | **CINAHL Search** |
|  | (MH "Osteoarthritis+") |
|  | TI (osteoarthritis OR arthritis OR joint degenerative disease OR Cartilage) OR AB (osteoarthritis OR arthritis OR joint degenerative disease OR Cartilage) |
|  | (MH "Diet") |
|  | TI Diet* OR AB Diet* |
|  | (MM "Cytokines”) OR (MM "Inflammation") OR (MH "Quality of Life") |
|  | TI (quality of life OR wellbeing OR weight* OR Symptoms OR Inflame* OR CRP OR C-reactive protein OR interleukin-1 OR interleukin-6 OR Tumour Necrosis Factor alpha OR IL-1α OR Cytokines OR pain) OR AB (quality of life OR wellbeing OR weight* OR Symptoms OR Inflame* OR CRP OR C-reactive protein OR interleukin-1 OR interleukin-6 OR Tumour Necrosis Factor alpha OR IL-1α OR Cytokines OR pain) |
