# Supplementary material for: The effectiveness of dietary intervention in osteoarthritis management: a systematic review and meta-analysis of randomized clinical trials
Source: Eur J Clin Nutr. 2025 Apr 28;79(10):959–71. doi: 10.1038/s41430-025-01622-0 (PMC12537491; doi:10.1038/s41430-025-01622-0)
Supplement: Supplementary file 2 — Appendix 2. [file 41430_2025_1622_MOESM2_ESM.docx]

**Appendix 2:** List of excluded studies.

| **Reasons of excluding** | **Title of excluded studies** |
| --- | --- |
| Wrong intervention | Precision Medicine Approach to Develop and Internally Validate Optimal Exercise and Weight-Loss Treatments for Overweight and Obese Adults With Knee Osteoarthritis: Data From a Single-Center Randomized Trial. |
| Wrong intervention | Effect of weight maintenance on symptoms of knee osteoarthritis in obese patients: a twelve-month randomized controlled trial. |
| Wrong intervention | Effects of exercise and weight loss on interstitial matrix turnover and tissue inflammation biomarkers in adults with knee osteoarthritis: the intensive diet and exercise for arthritis trial (IDEA). |
| Wrong intervention | The intensive diet and exercise for arthritis trial (IDEA): 18-month radiographic and MRI outcomes |
| Wrong intervention | Influence of weight loss, body composition, and lifestyle behaviors on plasma adipokines: A randomized weight loss trial in older men and women with symptomatic knee osteoarthritis |
| Abstract only | Cartilage loss during symptomatic maintenance after a clinically significant weight loss in obese osteoarthritis patients: A randomized controlled trial |
| Wrong intervention | Effect of Diet and Exercise on Knee Pain in Patients With Osteoarthritis and Overweight or Obesity: A Randomized Clinical Trial. |
| Abstract only | Effects of dietary weight loss with and without exercise on fibrosis biomarkers in adults with knee osteoarthritis |
| Wrong intervention | Comparing two low-energy diets for the treatment of knee osteoarthritis symptoms in obese patients: a pragmatic randomized clinical trial. |
| Protocol | Effect of liraglutide on body weight and pain in patients with overweight and knee osteoarthritis: protocol for a randomised, double-blind, placebo-controlled, parallel-group, single-centre trial. |
| Not RCT | Association Between Weight Loss and Spontaneous Changes in Physical Inactivity in Overweight/Obese Individuals With Knee Osteoarthritis: An Eight-Week Prospective Cohort Study. |
| Not RCT | Dose response relationship between weight loss and improvement in quality of life in persons with symptomatic knee osteoarthritis |
| Not RCT | A Low-Carbohydrate Diet To Reduce Self-Reported Pain In Non-Hispanic Black Women With Knee Osteoarthritis |
| Wrong intervention | Influencia de una dieta modificada en la calidad de vida en mujeres con obesidad y artrosis de rodilla antes de la cirugía ortopédica. |
| Not RCT | Effect of a modified hypocaloric diet on quality of life of obese patients with osteoarthritis |
| Not RCT | The effects of exercise and weight loss in overweight patients with hip osteoarthritis: Design of a prospective cohort study |
| Wrong intervention | Fit and Strong! Plus: Twelve and eighteen month follow-up results for a comparative effectiveness trial among overweight/obese older adults with osteoarthritis |
| Wrong intervention | Effects of intensive diet and exercise on knee joint loads, inflammation, and clinical outcomes among overweight and obese adults with knee osteoarthritis: the IDEA randomized clinical trial. |
| Protocol | Effectiveness of an anti-inflammatory diet versus low-fat diet for knee osteoarthritis: the FEAST randomised controlled trial protocol |
| Abstract only | CLINICAL CHANGES IN KNEE OSTEOARTHRITIS (KOA) PATIENTS  EXPOSED TO AN ANTI-INFLAMMATORY (ITIS)-DIET |
| Abstract only | The efficacy of a Mediterranean type diet on symptoms of osteoarthritis - A pilot study |
| Wrong intervention | Effects of adding a diet intervention to exercise on hip osteoarthritis pain: protocol for the ECHO randomized controlled trial. |
| Not RCT | Effects of an intensive weight loss program on knee joint loading in obese adults with knee osteoarthritis. |
| Protocol | The Intensive Diet and Exercise for Arthritis (IDEA) trial: design and rationale. |
| Wrong intervention | Liraglutide after diet-induced weight loss for pain and weight control in knee osteoarthritis: a randomized controlled trial. |
| Not RCT | The effect of weight loss on knee joint loading in obese knee osteoarthritis patients |
| Wrong Intervention | The independent and combined effects of intensive weight loss and exercise training on bone mineral density in overweight and obese older adults with osteoarthritis. |
| Wrong Intervention | Cost-Effectiveness of Telehealth-Delivered Exercise and Dietary Weight Loss Programs for Knee Osteoarthritis Within a Twelve-Month Randomized Trial. |
| Not RCT | CHANGE IN ULTRASOUND-BASED KNEE JOINT INFLAMMATORY MARKERS AFTER WEIGHT LOSS IN PATIENTS WITH OSTEOARTHRITIS: A PROSPECTIVE COHORT STUDY |
| Not RCT | Effect of a 16 weeks weight loss program on osteoarthritis biomarkers in obese patients with knee osteoarthritis: A prospective cohort study |
| Not RCT | Diet and Exercise and Knee Pain in Patients With Osteoarthritis and Overweight or Obesity. |
| Not RCT | Critically appraised paper: In overweight and obese adults with knee osteoarthritis, the addition of telehealth-delivered exercise and diet programs to online education improves pain and function [commentary]. |
| Abstract only | Change of diet and exercise improve knee pain only slightly in overweight patients with osteoarthritis |
| Wrong intervention | LONG-TERM EFFECTIVENESS OF A LIFESTYLE PROGRAM FOR OSTEOARTHRITIS: ONE-YEAR FOLLOW-UP OF THE PLANTS FOR JOINTS RANDOMIZED CLINICAL TRIAL |
| Wrong intervention | Effectiveness of Intensive Diet and Exercise on Knee Pain among Communities with Knee Osteoarthritis, Overweight, and Obesity: The WE-CAN Pragmatic Randomized Clinical Trial |
| Wrong Outcome | Effect of an exercise and dietary intervention on serum biomarkers in overweight and obese adults with osteoarthritis of the knee. |
| Wrong intervention | The Intensive Diet and Exercise for Arthritis (IDEA) trial: 18-month radiographic and MRI outcomes. |
| Wrong intervention | Effects of weight-loss on patellofemoral loading in overweight and obese adults with patellofemoral osteoarthritis: Secondary analysis from the idea randomized trial |
| Wrong intervention | Effect of a Lifestyle Program Based on a Whole Food Plant-based Diet, Physical Activity, and Stress Management in Patients with Metabolic Syndrome-Associated Osteoarthritis: A Randomized Controlled Trial |
| Abstract only | The assessment of a group of patients with knee osteoarthritis and obesity from Oradea Romania Using Short Form 36 |
| Not RCT | Influence of a meal-replacement diet on quality of life in women with obesity and knee osteoarthritis before orthopedic surgery. |
| Protocol | Train High Eat Low for Osteoarthritis study (THE LO study): protocol for a randomized controlled trial. |
| Wrong intervention | Changes in physical activity during a one-year weight loss trial with liraglutide in participants with knee osteoarthritis: secondary analyses of a randomised trial |
| Not RCT | An anti-inflammatory diet intervention for knee osteoarthritis: a feasibility study. |
| Wrong intervention | Glucosamine and chondroitin sulphate supplementation along with diet therapy provides better symptomatic relief in osteoarthritic patients as compared to diet therapy alone |
| Not RCT | Pilot study: Does a mediterranean diet-based weight loss programme improve health outcomes in overweight older women with knee osteoarthritis? |
| Wrong intervention | Changes in joint loads, leptin, and mmp-3 subsequent to long-term intensive weight loss and exercise: Secondary outcomes from the intensive diet and exercise for arthritis randomized clinical trial |
|  |  |
|  |  |
