# Supplementary material for: The effectiveness of dietary intervention in osteoarthritis management: a systematic review and meta-analysis of randomized clinical trials
Source: Eur J Clin Nutr. 2025 Apr 28;79(10):959–71. doi: 10.1038/s41430-025-01622-0 (PMC12537491; doi:10.1038/s41430-025-01622-0)
Supplement: Supplementary file 4 — Appendix 4 [file 41430_2025_1622_MOESM4_ESM.docx]

**Appendix 4:** Leave-One-Out Sensitivity Analyses for Pain, Physical Function, and Weight Outcomes.

| Leave-One-Out Sensitivity Analyses for Pain.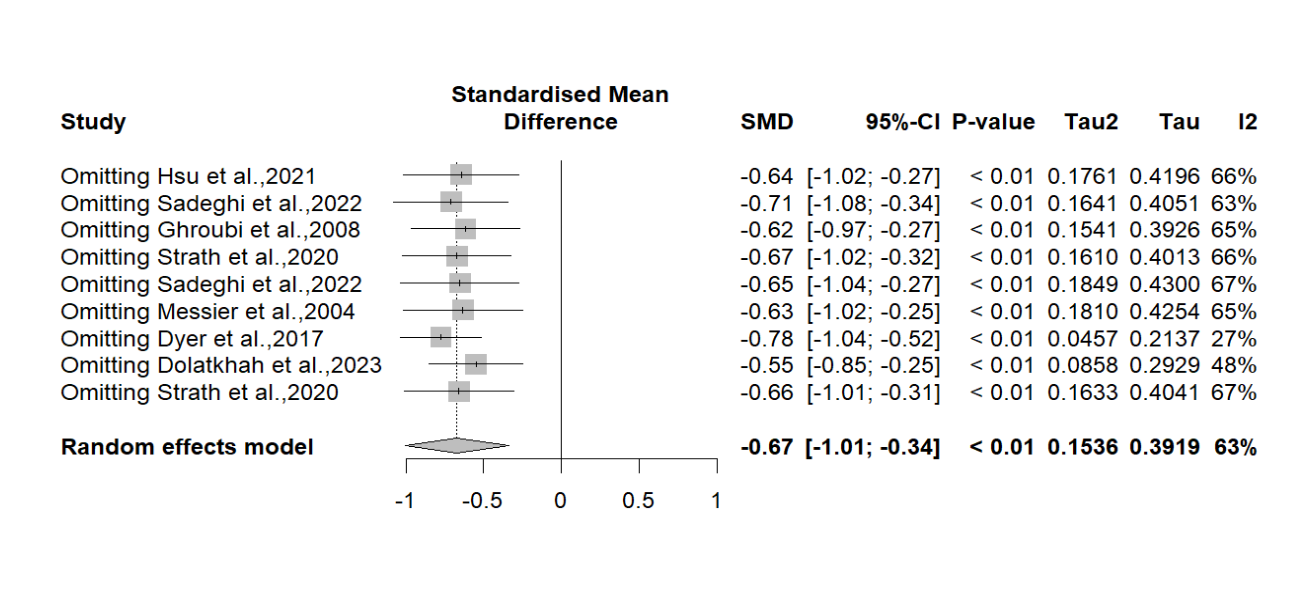 |
| --- |
| Leave-One-Out Sensitivity Analyses for Physical Function. 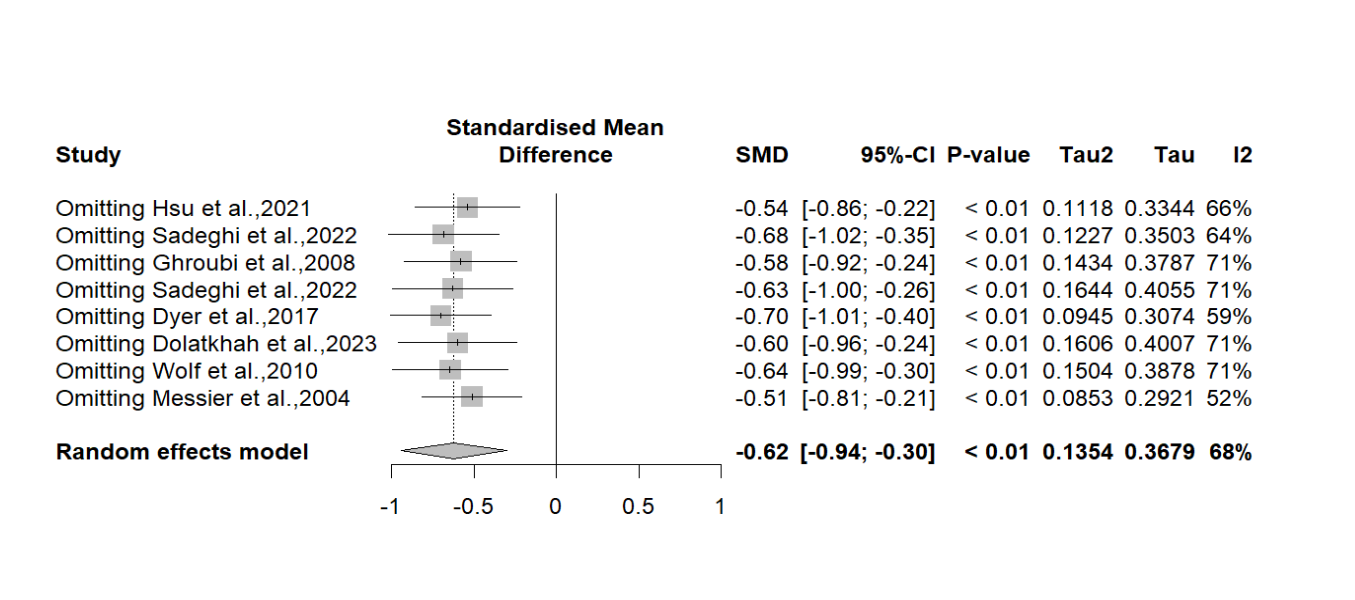 |
| Leave-One-Out Sensitivity Analyses for Weight.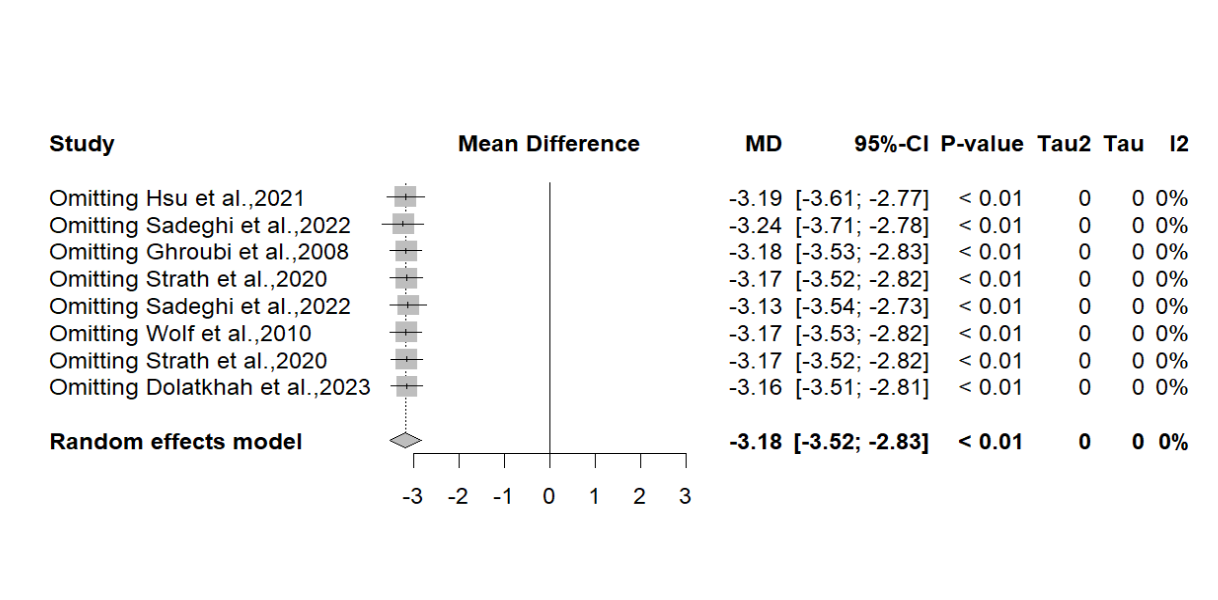 |
